# Development of PVDF Ultrafiltration Membrane with Zwitterionic Block Copolymer Micelles as a Selective Layer

**DOI:** 10.3390/membranes9080093

**Published:** 2019-08-01

**Authors:** Hajeeth Thankappan, Gauthier Bousquet, Mona Semsarilar, Antoine Venault, Yung Chang, Denis Bouyer, Damien Quemener

**Affiliations:** 1 IEM, Univ Montpellier, CNRS, ENSCM, 34095 Montpellier, France; 2 R&D Center for Membrane Technology, Department of Chemical Engineering, Chung Yuan Christian University, Chung-Li, Taoyuan 32023, Taiwan

**Keywords:** block copolymer, zwitterion, self-assembly, nanostructured membranes

## Abstract

In recent years, block copolymer micellar assemblies with the formation of structured nanoparticles have been considered as an emerging technology in membrane science. In this work, the poly(methyl methacrylate)-block-poly(sulfobetaine methacrylate) copolymer was directly synthesized using Reversible Addition-Fragmentation chain Transfer (RAFT) polymerization and self-assembled in a selective medium (2,2,2-trifluroethanol/water). Then, poly(methyl methacrylate)-block-poly(sulfobetaine methacrylate) copolymers were casted onto a commercial PVDF membrane to form a thin porous selective layer. The prepared nanoparticles and the resulting membranes were fully characterized using microscopy methods (SEM and AFM), whereas the membrane performance was evaluated in terms of permeability and the molecular weight cut off. The results from this study demonstrate the preparation of an ultrafiltration membrane made from the assembly of poly(methyl methacrylate)-block-poly(sulfobetaine methacrylate) copolymer micelles on the top of a PVDF membrane in the form of thin film. The copolymer chain orientation leads to a membrane surface enriched in hydrophilic PSBMA, which confers a suitable behavior for aqueous solution filtration on the membrane, while preserving the high chemical and mechanical resistance of the PVDF.

## 1. Introduction

In the past few decades, polymer membranes have attracted considerable attention in the separation of valuable compounds. Although a high diversity of polymer filtration membranes is now available, their broader use is still confronted to their limitations in terms of selectivity while maintaining a high permeability [1,2,3,4]. Self-assembly of well-defined amphiphilic block copolymers (BCPs) is increasingly viewed as a key strategy for preparing high performance porous materials gathering all the properties required for current and future technologies: fouling resistance, high permeability, high selectivity and a simple scale up manufacturing [5,6]. Under specific solvent conditions providing a selectivity among the polymer blocks, BCPs can self-assemble into various nanostructures with a domain spacing that depends on energetic repulsion and entropy loss [7,8]. The capability to engineer nanoporous membranes from amphiphilic block copolymers was highlighted in various practical applications like ultrafiltration and nano lithography [9,10,11].

In addition to concerns regarding permselectivity, a fine tuning of the membrane surface properties is usually required to optimize the ageing (leaching of additives), especially when the final application requires hydrophobic polymers as a membrane precursor on account of their superior chemical resistance. For the past few decades, poly(ethylene glycol) (PEG) has been massively used as a hydrophilic modifier for improving the surface wettability of hydrophobic membranes [12,13]. This intensive application went along with more thorough studies, concluding on a sensitivity towards oxidation leading to a change in the properties overtime, as well as a progressive releasing of the PEG additive in the surrounding aqueous medium due to a lack of anchoring strategy [14,15]. Zwitterionic polymers, poly(sulfobetaine methacrylate) (PSBMA) in particular, is another polymer family known for improving hydrophilicity, as well as providing non-specific protein resistance and high blood compatibility to hydrophobic solid materials [16,17,18,19]. Interestingly, zwitterionic polymers possessed a strong tendency to self-assemble, thanks to strong dipole–dipole interactions between the zwitterionic groups, and reinforced by a high Flory-Huggins interaction parameter between the hydrophobic and zwitterionic repeat units [20,21,22]. Their exceptional hydrophilicity has been used for instance to disrupt oil-in-water and water-in-oil emulsions thanks to a high water absorption [23]. However, more than their intrinsic antifouling properties, the polymer chain density on the membrane surface was also determined to strongly affect the antifouling properties of the membranes [19,24].

Reversible Addition-Fragmentation chain Transfer (RAFT) polymerization is one of the most widely applied reversible-deactivation radical polymerizations to synthesize the zwitterionic block copolymers. Many attempts were made to directly synthesize PSBMA block copolymers using RAFT polymerization techniques [25,26,27,28,29]. In all of the cases, the non-betaine block was hydrophilic. Therefore, direct synthesis of amphiphilic sulfobetaine-based block copolymer still represents a challenge due to the difficulty of selecting a solvent system that enables us to keep both parts solvated in the reaction medium. The main problem encountered is coming from the super ion hydration capacity of PSBMA, which makes it insoluble in the majority of organic solvents [30].

Numerous works have been reported using a post-polymerization approach to circumvent this drawback, whereby the corresponding 2-(dimethyl amino)ethyl methacrylate (DMAEMA) is polymerized in a suitable solvent and then subsequently modified with 1,3-propane sultone to yield PSBMA [31,32,33]. However, the post-polymerization approach has several disadvantages; (1) moderate conjugation efficiency due to steric hindrance of tertiary amine (2) non-negligible toxicity of the 1,3-propane sultone required for the modification, (3) long reaction time of typically several days, and more importantly (4) poorly-defined statistical copolymer of DMAEMA and SBMA depending on the conjugation yields. In general, the need for excess amount of 1,3-propane sultone and tedious purification steps resulting in low yields makes the post polymerization approach less feasible when large quantities are required. As an alternative, ionic liquids have been used as a solvent for polymerization of PBSMA amphiphilic block copolymers, although isolation and purification from ionic liquids proved to be difficult [34,35,36,37]. In that perspective, the use of fluorinated solvents such as 2,2,2-trifluoroethanol (TFE) appeared to be a better choice [38]. TFE was therefore selected as a candidate to maintain both zwitterionic and hydrophobic segments under good solvent conditions in order to prepare amphiphilic zwitterionic block copolymers in solution.

The main objective of this work is to examine the possibility of preparing an ultrafiltration membrane for aqueous solutions based on the poly(vinylidene difluoride) (PVDF) polymer in the presence of a block copolymer to impose other physico-chemical properties at the interfaces. While PVDF is known for its good chemical and thermal resistance, it is also a hydrophobic polymer, resulting in accentuated resistance of the corresponding porous materials to the water flow. In this work, we firstly report the synthesis of the amphiphilic block copolymer poly(methyl methacrylate)-block-poly(sulfobetaine methacrylate) (PMMA-*b*-PSBMA) using TFE, suitable for the polymerization of zwitterionic SBMA initiated in the presence of a PMMA chain transfer agent. PMMA was selected as a hydrophobic polymer compatible with PVDF, while PSBMA was selected as hydrophilic polymer to improve the surface hydrophilicity and anti-fouling properties of the PVDF membrane.

Self-assembly of block copolymers in a selective solvent is a promising approach to produce nanoscale particles with well-defined geometry, size, and functionality [39,40], Therefore the self-assembly behavior of the prepared PMMA-*b*-PSBMA block copolymers is secondly investigated in a selective solvent system. Then, PMMA-*b*-PSBMA copolymer micellar solution was casted on a commercial PVDF membrane to form a thin porous film. The PMMA block is susceptible to interact with PVDF membrane material, so that a strong anchoring of the micelles is expected via a simple coating process. The morphology and hydrophilicity of the block copolymer porous film were characterized by SEM, AFM and water contact angle. Finally, the pure water permeability and Molecular Weight Cut-Off (MWCO) of the membrane was estimated in a dead-end filtration setup.

## 2. Materials and Methods 

### 2.1. Materials

Methyl methacrylate (MMA), 4-cyano-4(phenylcarbonothioylthio) pentanoic acid, azobisisobutyronitrile, 2-(*N*-3-Sulfopropyl-*N*,*N*-dimethyl ammonium)ethyl methacrylate, toluene, methanol, methylene chloride, 2,2,2-trifluoroethanol, and tetrahydrofuran were purchased from Merck (Darmstadt, Germany) and were used as received. NMR solvents trifluoroethanol-d_3_ and CDCl_3_ were received respectively from Sigma-Aldrich and Eurisotop (Saint Aubin, France). Commercial PVDF membranes were purchased from Millipore (Durapore membrane filter, 0.1 µm).

### 2.2. Characterizations

#### 2.2.1. Nuclear Magnetic Resonance (NMR)

^1^H NMR spectra were acquired in either CDCl_3_ or trifluoroethanol-d_3_ using a Bruker 300 MHz spectrometer (Billerica, MA, USA).

#### 2.2.2. Scanning Electron Microscopy (SEM)

SEM analyses were performed using a Hitachi S-4500 instrument (Tokyo, Japan) operating at spatial resolution of 1.50 nm at 15 kV energy. The samples were dried and covered with an ultra-thin layer of electrically conductive Platinum deposited by evaporation under high vacuum. To get a SEM image of the membrane cross section, samples were carefully frozen in liquid nitrogen for 5 min before a clear cut was given.

#### 2.2.3. Atomic Force Microscopy (AFM)

AFM images were obtained using a Pico SPM II provided by Molecular Imaging (Tempe, CA, USA), controlled by the Pico View 1.10 software. The experiments were all done in tapping mode. The tip used was PPS-FMR purchased from Nanosensors (Neuchatel, Switzerland) with a frequency resonance between 45–115 kHz and a constant force of 0.5–9.5 N/m. Gwyddion 2.25 software (Brno, Czech Republic) was used to process the images.

#### 2.2.4. Size-Exclusion Chromatography (SEC)

True average molecular weight of PMAA macro-CTA was determined using Size Exclusion Chromatography (SEC) performed with a triple detector array from Viscotek (TDA 305, Malvern instruments, Worcestershire, UK). The Viscotek SEC apparatus was equipped with two mixed-columns with a common particle size of 5 µm using THF as an eluent (1.0 mL/min). The Viscotek system contains a refractive index detector (RI, concentration detector), a four-capillary differential viscometer and light scattering. OmniSEC software was used for analysis and data acquisition.

#### 2.2.5. Dynamic Light Scattering (DLS)

DLS studies were performed using a Litesizer^TM^ 500 from Anton Paar (Graz, Austria). Samples were analyzed at 25 °C in quartz cuvettes.

### 2.3. Synthetic Procedure

#### 2.3.1. Synthesis of Poly(methyl methacrylate) Macro-Chain Transfer Agent (PMMA macro-CTA)

A typical synthesis of PMAA macro-CTA was performed as follows: methyl methacrylate (MMA; 12 g; 118 mmol), 4-cyano-4-(phenylcarbonothioylthio)pentanoic acid (23 mg; 0.079 mmol), and Azobisisobutyronitrile (6.5 mg; 0.039 mmol) was dissolved in toluene (12.0 g). The mixture was thoroughly purged with oxygen free nitrogen for 30 min and then immersed into an oil bath at 70 °C for 16 h. The polymerization was stopped by sudden cooling of the reaction mixture and subsequent exposure to the air. The above reaction mixture was diluted with some methylene chloride and precipitated into 10-fold excess methanol. The solid was dissolved in methylene chloride and precipitated again. The dissolution-precipitation procedure was repeated 3 times in total. The pale pink solid after precipitation was dried under vacuum for 24 h. (60% conversion as judged by ^1^H NMR spectroscopy in CDCl_3_).

#### 2.3.2. Synthesis of Poly(methyl methacrylate)-b-poly(sulfobetaine methacrylate) (PMMA-b-PSBMA) Block Copolymer

PMMA macro-CTA (2.7 g, 0.097 mmol), SBMA (8.1 g, 29 mmol), and AIBN (4 mg, 0.0243 mmol) were dissolved in 2,2,2-trifluoroethanol in a flask. The solution was degassed by purging with nitrogen for 30 min. The block copolymerization was performed at 70 °C for 16 h and quenched by cooling the solution in iced water. The polymer mixture was slowly added into a large excess of THF and methanol mixture (1:1) to obtain PMMA-*b*-PSBMA block copolymer. The solid was dissolved in 2,2,2-trifluoroethanol and precipitated again. The precipitation procedure was repeated 2 times. The block copolymer was dried under vacuum to a constant weight and characterized by ^1^H NMR spectroscopy. (62% conversion as judged by ^1^H NMR spectroscopy in trifluoroethanol-d_3_). 

### 2.4. Membrane Preparation and Characterization

For the preparation of the membrane, a block copolymer of PMAA-*b*-PSBMA (20% by weight) was casted on the PVDF hydrophobic membrane with a 250 μm casting knife. The membrane samples were dried under ambient conditions for 48 h. The prepared membrane was then cut with a disk shape (d = 2.5 cm) and placed into a 10 mL filtration cell (Amicon 8010 stirred cell). The cell was connected to a water reservoir and a compressed air line. The water permeability measurements were made at drop pressures going from 0.5 to 4.0 bar. The weight of water permeating across the membrane was recorded by the SartoConnect software at given time intervals. All the filtration experiments were carried out at room temperature with ultrapure dust-free water (filtered through a 400 μm filter).

### 2.5. Water Contact Angle (WCA)

Water Contact angles were conducted onto a GBX equipment (Digidrop, Romans, France). The given value is calculated from the average of at least ten independent measurements made from applying a water droplet of 3 μL to the surface. The contact angle was calculated using computerized image analysis.

### 2.6. Determination of MWCO

The Molecular weight cut-off (MWCO) of the membrane was estimated from the filtration of polyethylene glycol (PEG) aqueous solutions (concentration of 1g/L) with Mw of 10, 35, 100 and 300 kDa. The concentrations of PEG were measured by flow injection analysis using refractometer 2414 (Waters Corporation, Milford, MA, USA). The solution rejection (R) was calculated using the following equation Equation (1):

(1) $$R=1-C_{p}/C_{f}\times 100$$

where C_p_ is the concentration of PEG in permeate and C_f_ is the concentration of PEG in the feed. 

## 3. Results and Discussions

### 3.1. Preparation and Characterization of PMMA-b-PSBMA Block Copolymer

**Scheme 1:**
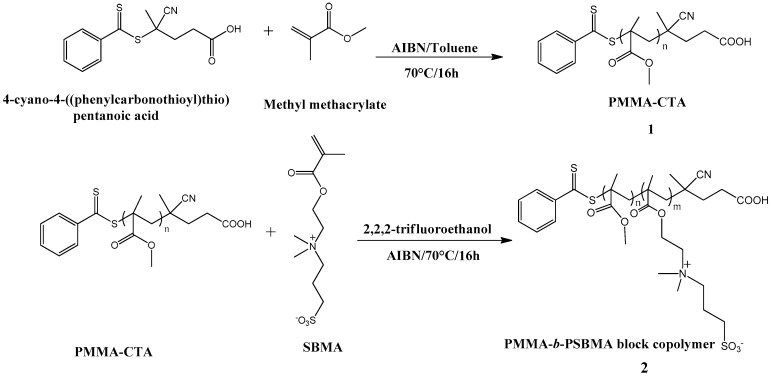
RAFT polymerization of PMMA-*b*-PSBMA block copolymer.

PMMA-*b*-PSBMA block copolymer has been prepared by RAFT polymerization as depicted in Scheme 1.

In a first step, a PMAA macro-CTA is synthesized in toluene at 70 °C (60% conversion, *M*_n_ = 23 kg·mol^−1^, Ð = 1.18, Figure S1). RAFT polymerization of SBMA was then performed at 70 °C using PMMA macro-CTA and AIBN in 2,2,2-trifluoroethanol (62% conversion, Figure S2). We first attempted to initiate the RAFT polymerization in different polar solvents like DMF, DMSO, and 1,4-dioxane, but the polymerization medium quickly turns turbid and a precipitate is formed due to the extremely low solubility of PSBMA growing chains (see solubility data, Table S1). To solve this problem, a highly polar fluorinated solvent, 2,2,2-trifluoroethanol (TFE), was used to ensure the solubility of the monomer and the polymer throughout the polymerization. Atom Transfer Radical Polymerization (ATRP) and RAFT polymerization of SBMA in TFE medium have been reported previously, producing well-controlled polymers [38,41]. Since TFE is also a good solvent for PMMA, the chain extension of PMMA macro-CTA was carried out in this solvent. ^1^H NMR spectrum in trifluoroethanol-d_3_ was used to evaluate Mn of PMMA-*b*-PSBMA block copolymer since no suitable solvent system compatible with Size-Exclusion Chromatography was found. By comparing the NMR signals from –O–CH_3_ (peak “a”) of PMMA and –N–CH_2_–C*H_2_* (peak “e”) from PSBMA (Figure S3), a Mn of 54 kg·mol^−1^ was estimated for the PSBMA block. 

**Figure 1:**
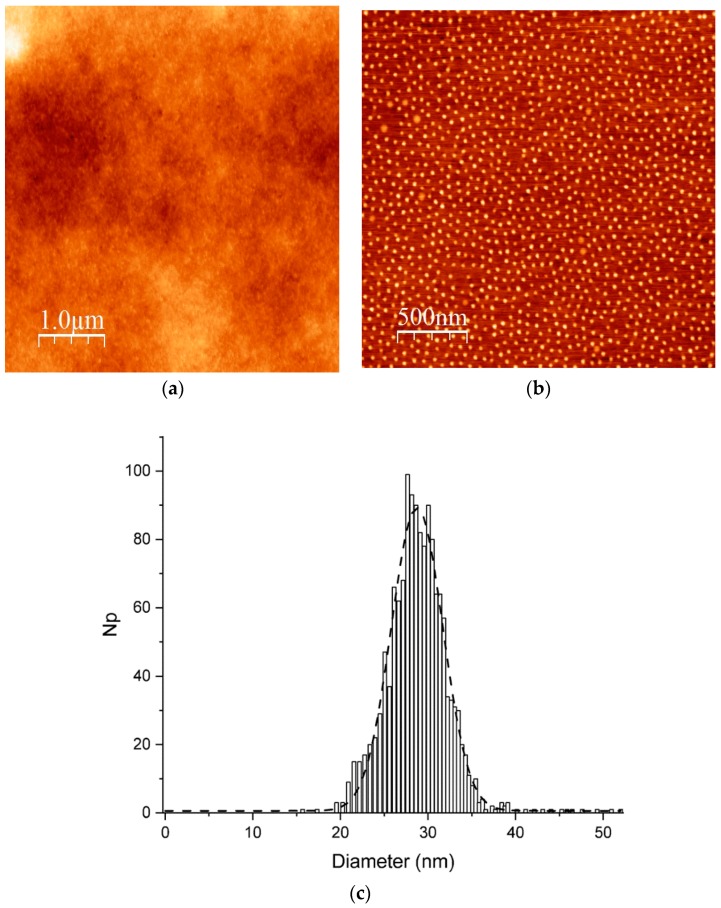
(**a**) AFM images of PMMA-*b*-PSBMA block copolymer in 2,2,2-trifluoroethanol coated on silicon wafer with 5% *w*/*w* polymer concentration, (**b**) PMMA-*b*-PSBMA block copolymer in 2,2,2-trifluoroethanol: water mixture 1:1 coated onto a silicon wafer with 5% *w*/*w* polymer concentration, (**c**) Particle size distribution of PMMA-*b*-PSBMA block copolymer built from the AFM image analysis.

The self-assembly behavior of PMMA-*b*-PSBMA block copolymer was then investigated in various solvents. Firstly, we prepared a solution of THF: water mixture 1:1 in volume by drop wise addition of water in a solution of PMMA-*b*-PSBMA block copolymer at 5 wt % in THF and let it stir for 8 h. Afterwards, lots of aggregates were found in the solution, thereby preventing any subsequent use. In the second trial, we prepared a solution of NMP: water mixture 1:1 in volume by addition of water at 60 °C to destabilize the aggregates but still lots of aggregates were observed. Similar results were obtained with a *N*,*N*-dimethylacetamide-water mixture. The micellization process from PMMA-*b*-PSBMA block copolymer is actually a problem due to the initial lack of PSBMA solubility in THF. Solutions were thus prepared in TFE with 5 wt % of PMMA-*b*-PSBMA block copolymer stirred for 1 h. The homogeneous solution was then drop casted onto silicon wafer and dried at 25 °C for 8 h. A thin and homogeneous film was obtained and analyzed using AFM. As expected, no micelles were observed in the film (Figure 1a), given the non-selective nature of this solvent. Micelles were then produced by dissolving the block copolymer (5 wt %) in TFE, followed by a drop wise addition of water until reaching a TFE: water ratio of 1:1 in volume over a period of 30 min at 60 °C. After stirring at 25 °C for 5 h, a clear solution with no aggregates was obtained and drop casted onto a silicon wafer. Isolated nanoparticles with a diameter of about 30 nm with narrow size distribution (Figure 1c) were observed by AFM (Figure 1b).

**Figure 2:**
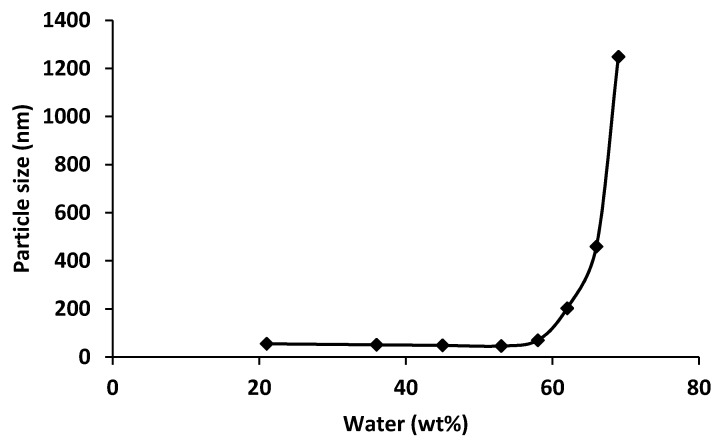
Particle size (nm) of PMMA-*b*-PSBMA block copolymer solution in 2,2,2-trifluoroethanol/water medium vs. water concentration.

The hydrodynamic diameter of PMMA-*b*-PSBMA block copolymer micelles was followed by DLS upon the addition of water. Starting from PMMA-*b*-PSBMA block copolymer at 1 mg/mL in TFE, water was slowly added and the solution was let to stir for 20 min before DLS measurement (Figure 2). Two water concentration regimes could be identified. Up to a water concentration of about 60 wt %, the hydrodynamic size remained stable around 55 nm. However, above 60% water, a sharp increase of the size was observed and visible aggregates were formed in the solution. Although this destabilization remains unclear to us, it underlines a range of water concentrations to be respected in order to produce stable block copolymer micelles. 

### 3.2. Preparation and Characterization of Nanostructured Membranes

**Figure 3:**
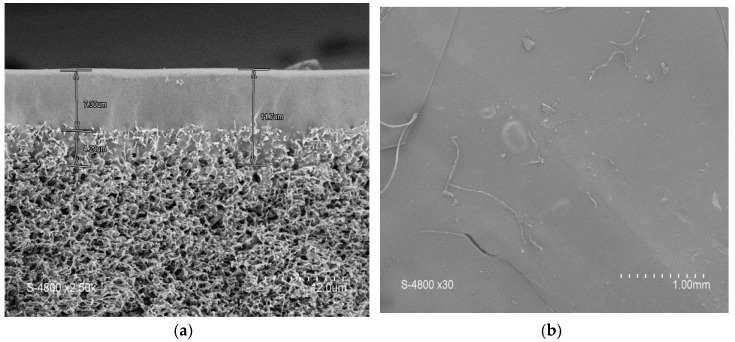
(**a**) SEM images of a membrane obtained from a micellar solution of 20 wt % of PMMA-b-PSBMA block copolymer solution in TFE:water mixture (87:13% in volume) casted on the surface of a commercial hydrophobic PVDF membrane using a 250 μm casting knife. (**a**) Cross section. (**b**) Top surface.

To prepare a porous film, a micellar solution of 20 wt % of PMMA-*b*-PSBMA block copolymer solution in TFE:water mixture (87:13% in volume) was casted on the surface of a commercial hydrophobic PVDF membrane using a 250 μm casting knife. The membrane was then characterized by SEM after 48 h of drying at 25 °C (Figure 3). 

**Figure 4:**
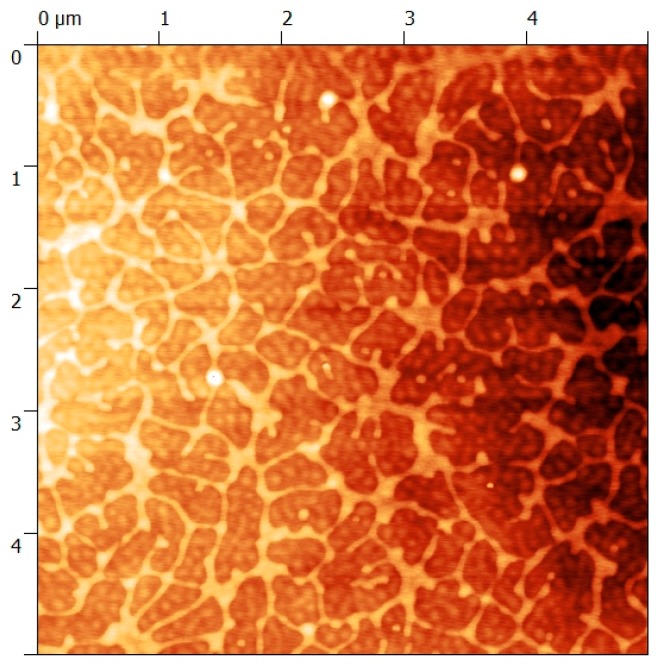
AFM image of a membrane obtained from a micellar solution of 20 wt % of PMMA-b-PSBMA block copolymer solution in TFE:water mixture (87:13% in volume) casted on the surface of a commercial hydrophobic PVDF membrane using a 250 μm casting knife.

As seen in Figure 3, the top surface is completely covered by the block copolymer film. It has to be noted that the small cracks on the surface of the film have appeared during the SEM sample preparation and do not reflect potential defects on the membrane. The partial penetration of the block copolymer layer in the PVDF membrane, as observed in the cross section (Figure 3a) ensured a high cohesion of the coating during the filtration process. The thickness of the superficial block copolymer coating was about 7 µm. Additionally, the AFM image in Figure 4 enables us to confirm the presence of spherical micelles with a diameter of about 65 nm. A network of partially fused micelles can also be seen. These partially fused micelles could have been formed as a result of the solvent composition change during the drying step. TFE evaporates faster than water, leading to a progressive increase of the water concentration overtime. This water enriched medium could lead to partial aggregation (as discussed previously the PMMA-*b*-PSBMA micelles aggregate in water rich environment- Figure 2) and to the formation of islands as observed in Figure 4. A water contact angle (WCA) of 65° was measured on the top surface, which confirms the hydrophilic nature of the thin porous film.

The prepared membrane was placed in a dead-end filtration setup and filled with ultrapure water. The drop pressure has been gradually increased from 0 to 4 bar. It has to be noted that a conditioning step of 30 min was respected before any data collection occurred. 

**Figure 5:**
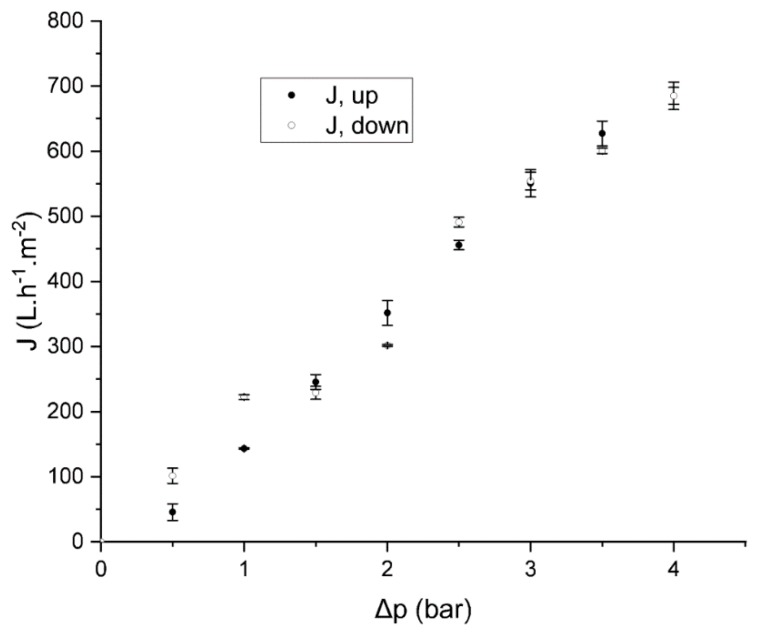
Water flux (J) as a function of the pressure drop when increasing (J, up) and decreasing (J, down) the water pressure of a membrane obtained from a micellar solution of 20 wt % of PMMA-b-PSBMA block copolymer solution in TFE:water mixture (87:13% in volume) casted on the surface of a commercial hydrophobic PVDF membrane using a 250 μm casting knife.

**Figure 6:**
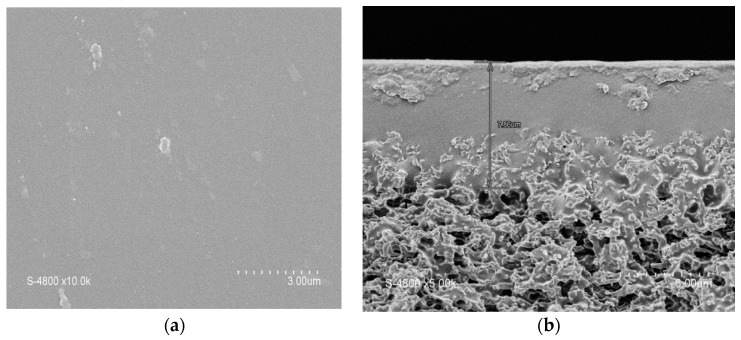
SEM images of the block copolymer porous film casted on the surface of the hydrophobic PVDF membrane after water filtration. (**a**) Top surface. (**b**) Cross section.

The membrane showed a steady increase in flux as the pressure increased (Figure 5) with a permeability (Lp) of 188 L·h^−1^·m^−2^·bar^−1^. No significant hysteresis was found when measuring the water flux while decreasing the pressure (Lp = 170 L·h^−1^·m^−2^·bar^−1^). The stability of the block copolymer layer was further confirmed by SEM after water permeation (Figure 6), which showed the membrane’s integrity.

**Figure 7:**
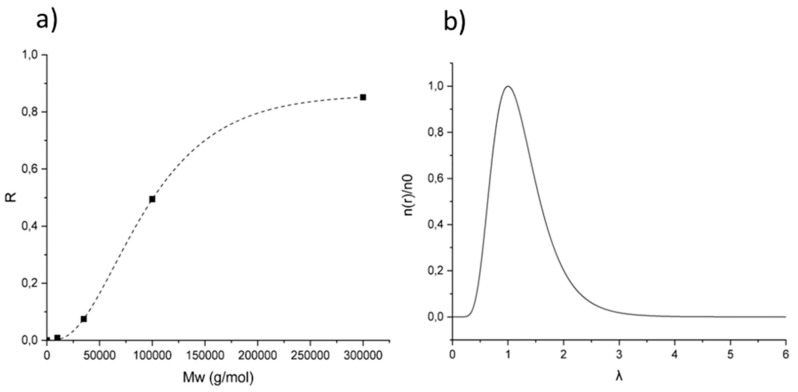
(**a**) Polyethylene glycol (PEG) rejection curve: rejection coefficient (R) vs. the molecular weight of PEG. (**b**) Log-normal pore size distribution: reduced number of pores per unit of area n(r)/n_0_ vs. λ, the ratio of solute radius to pore radius.

An estimation of the pore size of the membrane can be given based on the separation data obtained from filtration experiments using polyethylene glycol (PEG) of different molecular weights. The solute rejection versus the solute diameter is plotted with a log-normal model (Figure 7a). Using the Einstein–Stokes radius (ESr) of the solute, the mean pore size can then be considered as the solute diameter that corresponds to a retention of 50%, whereas the geometric standard deviation can be obtained from the ratio of solute diameter at retentions of 84.13% and 50% [42]. Here, the calculated pore diameter is thus 25.9 ± 1.7 nm. Based on these values, the pore size distribution was plotted in Figure 7b by using a log-normal model [43].

## 4. Conclusions

In summary, PMMA-*b*-PSBMA block copolymer were synthesized using a RAFT polymerization synthesis at 70 °C in 2,2,2,-trifluroethanol and then self-assembled in a selective medium (2,2,2,-trifluroethanol/water). Micelles were formed and characterized by DLS (D_h_ = 55 nm) and AFM (D = 30 nm). The block copolymer micellar solution was then casted onto the top of a commercial PVDF membrane to form a hydrophilic selective layer. SEM and AFM analysis confirmed the formation of a homogeneous micelle coating with a thickness of about 7 μm. Water permeation test with drop pressures up to 4 bars was conducted without any deterioration of the membrane. Filtration tests show the stability of the nanoporous membranes under a range of applied pressures (0–4 bars). In addition, the membrane pore size was evaluated based on MWCO determined by permeation of polyethylene glycol (PEG) having different molecular weights. The mean effective pore size of the membrane was about 26 nm. Future works will focus on the application of these materials as ultrafiltration membranes and the presence of zwitterions at the interface could open perspectives in anti-fouling technologies, such as required in biomedical applications.

## Supplementary Materials

The following are available online at https://www.mdpi.com/2077-0375/9/8/93/s1, Figure S1: ^1^H NMR spectra of crude mixture of PMAA macro-CTA in CDCl_3;_ Figure S2: ^1^H NMR spectra of PMMA-*b*-PSBMA block copolymer crude mixture in trifluoroethanol-d_3_; Figure S3: A) ^1^H NMR spectra of PMAA macro-CTA in CDCl3, B) PMMA-*b*-PSBMA block copolymer in trifluoroethanol-d_3_ C) GPC chromatogram of PMMA macro-CTA; Table S1: Solubility data SBMA and PMMA Macro CTA.
