# Supplementary material for: Development of PVDF Ultrafiltration Membrane with Zwitterionic Block Copolymer Micelles as a Selective Layer
Source: Membranes (Basel). 2019 Aug 1;9(8):93. doi: 10.3390/membranes9080093 (PMC6723104; doi:10.3390/membranes9080093)
Supplement: Supplementary file 1 [file membranes-09-00093-s001.pdf]

membranes

Article

Supplementary Materials: Development of PVDF Ultrafiltration Membrane with Zwitterionic Block Copolymer Micelles as a Selective Layer

Hajeeth Thankappan [1], Gauthier Bousquet [1], Mona Semsarilar [1], Antoine Venault [2], Yung Chang [2], Denis Bouyer [1] and Damien Quemener [1,*]

[1] IEM, Univ Montpellier, CNRS, ENSCM, 34095 Montpellier, France
[2] R&D Center for Membrane Technology, Department of Chemical Engineering, Chung Yuan Christian University, Chung-Li, Taoyuan 32023, Taiwan
* Correspondence: damien.quemener@umontpellier.fr

Received: 11 July 2019; Accepted: 26 July 2019; Published: 1 August 2019

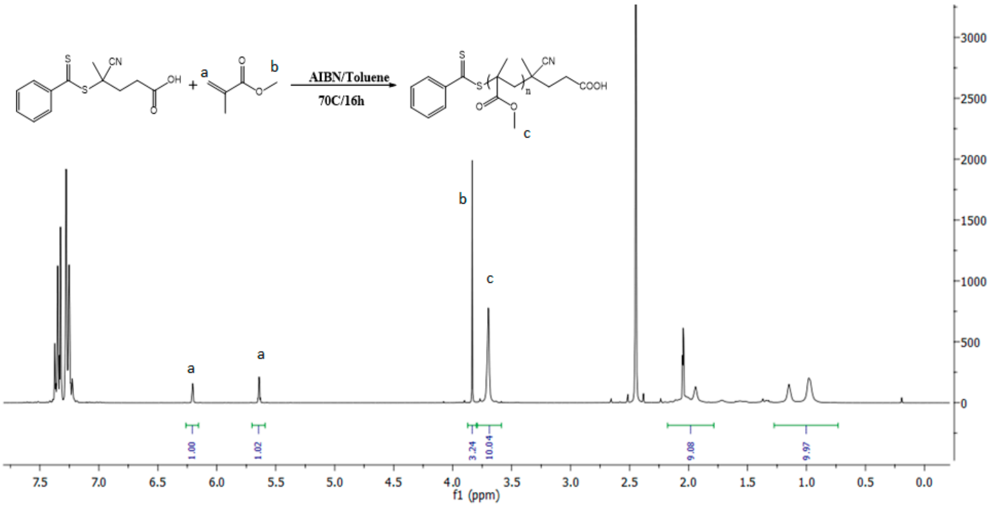

Figure S1. ^{1}H NMR spectra of crude mixture of PMAA macro-CTA in $CDCl_3$.

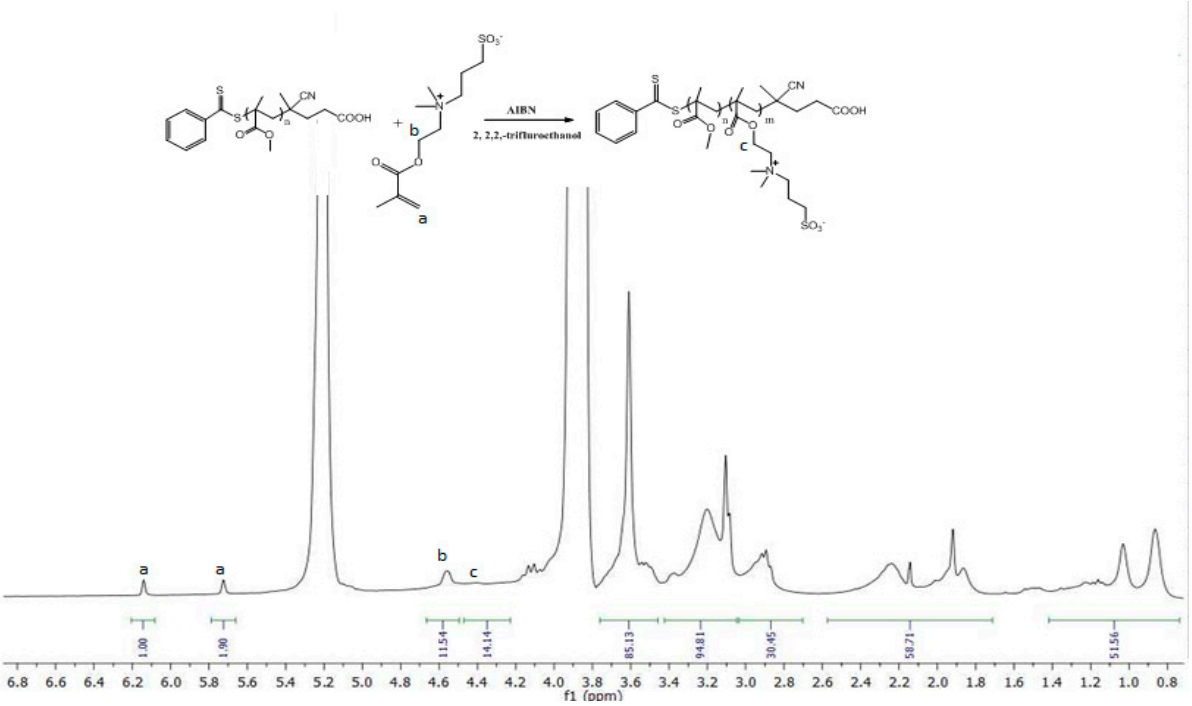

Figure S2. ^{1}H NMR spectra of PMMA-*b*-PSBMA block copolymer crude mixture in trifluoroethanol-d_3.

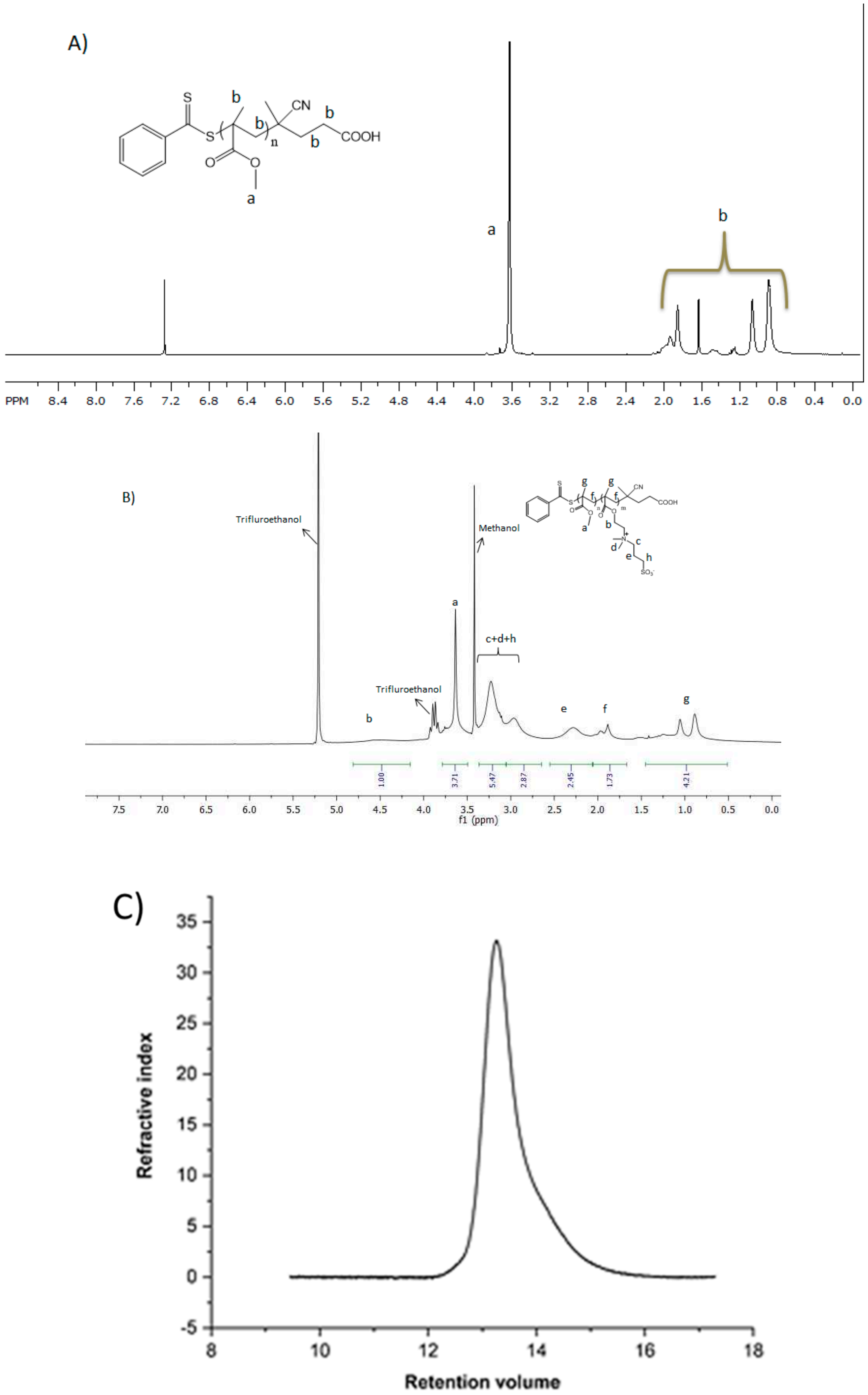

Figure S3. **A**) ^{1}H NMR spectra of PMAA macro-CTA in CDCl3, **B**) PMMA-*b*-PSBMA block copolymer in trifluoroethanol-d_3. **C**) GPC chromatogram of PMMA macro-CTA.

Table S1. Solubility data SBMA and PMMA Macro CTA.

Solvent	SBMA Monomer	PMMA Macro - CTA
Water	Soluble	Insoluble
Ethanol	Soluble	Insoluble
DMF	Insoluble	Soluble
Toluene	Insoluble	Soluble
THF	Insoluble	Soluble
1,4-dioxane	Insoluble	Soluble
Acetonitrile	Insoluble	Soluble
DMSO	Insoluble	Soluble
2,2,2-trifluoroethanol	Soluble	Soluble
